# Assessment of transmissibility and measures effectiveness of SARS in 8 regions, China, 2002-2003

**DOI:** 10.3389/fcimb.2023.1212473

**Published:** 2023-08-10

**Authors:** Jia Rui, Huimin Qu, Shuo Zhang, Hong Liu, Hongjie Wei, Buasiyamu Abudunaibi, Kangguo Li, Yunkang Zhao, Qiao Liu, Kang Fang, Laurent Gavotte, Roger Frutos, Tianmu Chen

**Affiliations:** ^1^ State Key Laboratory of Molecular Vaccinology and Molecular Diagnostics, School of Public Health, Xiamen University, Xiamen, Fujian, China; ^2^ Espace-Dev, Université de Montpellier, Montpellier, France; ^3^ French Agricultural Research Centre for International Development (CIRAD), URM 17, Intertryp, Montpellier, France

**Keywords:** SARS, transmissibility, control measures, basic reproduction number, effective reproduction number, time-varying reproduction number

## Abstract

**Background:**

Severe acute respiratory syndrome (SARS) is a form of atypical pneumonia which took hundreds of lives when it swept the world two decades ago. The pathogen of SARS was identified as SARS-coronavirus (SARS-CoV) and it was mainly transmitted in China during the SARS epidemic in 2002-2003. SARS-CoV and SARS-CoV-2 have emerged from the SARS metapopulation of viruses. However, they gave rise to two different disease dynamics, a limited epidemic, and an uncontrolled pandemic, respectively. The characteristics of its spread in China are particularly noteworthy. In this paper, the unique characteristics of time, space, population distribution and transmissibility of SARS for the epidemic were discussed in detail.

**Methods:**

We adopted sliding average method to process the number of reported cases per day. An SEIAR transmission dynamics model, which was the first to take asymptomatic group into consideration and applied indicators of *R*_0_, *R_eff_*, *R_t_* to evaluate the transmissibility of SARS, and further illustrated the control effectiveness of interventions for SARS in 8 Chinese cities.

**Results:**

The *R*_0_ for SARS in descending order was: Tianjin city (*R*_0_ = 8.249), Inner Mongolia Autonomous Region, Shanxi Province, Hebei Province, Beijing City, Guangdong Province, Taiwan Province, and Hong Kong. *R*_0_ of the SARS epidemic was generally higher in Mainland China than in Hong Kong and Taiwan Province (Mainland China: *R*_0_ = 6.058 ± 1.703, Hong Kong: *R*_0_ = 2.159, Taiwan: *R*_0_ = 3.223). All cities included in this study controlled the epidemic successfully (*R_eff_<*1) with differences in duration. *R_t_* in all regions showed a downward trend, but there were significant fluctuations in Guangdong Province, Hong Kong and Taiwan Province compared to other areas.

**Conclusion:**

The SARS epidemic in China showed a trend of spreading from south to north, i.e., Guangdong Province and Beijing City being the central regions, respectively, and from there to the surrounding areas. In contrast, the SARS epidemic in the central region did not stir a large-scale transmission. There were also significant differences in transmissibility among eight regions, with *R_0_* significantly higher in the northern region than that in the southern region. Different regions were able to control the outbreak successfully in differences time.

## 1 Introduction

Severe acute respiratory syndrome (SARS), a type of atypical pneumonia caused by the pathogen of SARS-coronavirus (SARS-CoV). An outbreak of SARS occurred from 2002 to 2003, with the cumulative number of cases worldwide exceeding 8,000, and most of the infections were concentrated in China (more than 7,000 cases). The virus had spread to more than 20 provinces and regions in China, and had great impact on the psychological behavior of the general public (Shi Kan et al, 2003). Although the SARS epidemic has ended, SARS and other homologous coronaviruses such as COVID-19, Middle East respiratory syndrome (MERS) etc. still have the potential threat of re-outbreak and re-epidemic due to the similar characteristics such as rapid onset, high transmissibility, and multiple transmission routes. SARS-CoV and SARS-CoV-2 both belong to the SARS metapopulation of viruses from which they emerged at different time and most likely different places. However, despite being closely related and belonging to the same metapopulation, these two viruses caused diseases characterized by totally different dynamics. COVID-19 caused by SARS-CoV-2 is a pandemic very difficult to control whereas SARS, caused by SARS-CoV, was a limited epidemic which was efficiently controlled. It is important to understand the difference of dynamic. Therefore, it is critical for researchers to attach great attention to study the dynamic of the SARS outbreak. Importantly, the experience of SARS prevention and control can be applied to other emerging infectious diseases as well.

Most of the previous studies focused on the virology, origin, epidemiology, clinical features, as well as the socioeconomic impact of SARS epidemic and the assessment of SARS transmissibility. Studies also have been conducted to evaluate the transmissibility of SARS-CoV. Mathematical models, such as stochastic population dynamic models, individual-based models, epidemic curve fitting, and Markov Chain Monte Carlo (MCMC) combined with Bayesian models, was also applied in previous researches, to explore and analyze the transmission of SARS in Hong Kong, Singapore, Vietnam, Canada, and other regions. Basic reproduction number (*R*_0_) was 2.7 in the early stage of SARS in Hong Kong (Riley et al., 2003), while it was estimated by various models that the transmission of SARS in Hong Kong ranged from 2 to 4 (Riley et al., 2003; Fraser et al., 2004; Wallinga and Teunis, 2004; Lekone, 2008). The effective reproduction number (*R_eff_*) was also estimated to be 0.001 during the control of SARS epidemic in Hong Kong (Lekone, 2008). In Singapore, estimation for *R*_0_ was around 3 when there was no intervention (Lipsitch et al., 2003; Wallinga and Teunis, 2004). Although the existence of super-communicator individuals was suspected, this was mostly linked to the fact that the virus had different modes of transmission rather than to the human host itself (Masuda et al., 2004). The average *R_eff_* in Vietnam and Canada were 2.4 and 2.7, respectively, which were consistent with data from Hong Kong and Singapore (Wallinga and Teunis, 2004). Modeling studies have included population and individual characteristics, confirming that the estimated *R*_0_ for SARS in different regions ranged from 1 to 5 (Hethcote, 2000a; Lloyd-Smith et al., 2003), and due to the regional heterogeneity, the evaluation for *R_0_* is different. In addition, some studies (Wilder-Smith et al., 2005; Tan, 2006; Tuan et al., 2007) have shown that asymptomatic individuals are also infectious, but the above models do not consider the transmission of asymptomatic group in the population, which may lead to an underestimation of the true transmissibility of SARS.

There are relatively comprehensive studies on SARS in Hong Kong, Taiwan, and Singapore. However, a comprehensive comparative analysis of the SARS epidemic in mainland China is still not available. Mathematical modelling is a relatively novel epidemiological research method allowing for simulation to describe the potential mechanisms, clarify the dynamics of disease development, population transmission of infected individuals, and assess the transmissibility of SARS in different periods (Anderson, 1991; Donnelly et al., 2004). Therefore, we constructed a transmission dynamics model that includes asymptomatic infections, and applied it to the SARS transmission route to evaluate and compare the transmissibility of SARS in Mainland China with more severe epidemic. Therefore, in this study, we have analyzed the reasons for the differences in transmissibility in the different areas, summarized the decrease in transmissibility, and finally, drew conclusions to be used for the current transmission control of the COVID-19 and novel coronavirus in the future.

## 2 Methods

### 2.1 Data collection

A dataset was built and mainly included reported date of global SARS cases derived from WHO (Organization, 2015). The temporal distribution of SARS cases in various provinces and regions of China and the information obtained on individual cases were obtained from the literature and dataset (Jia et al., 2009; P., 2019).

### 2.2 Statistical methods

To minimize interference with noise and to ensure *R_t_* calculation validity due to centralized reporting and processing of WHO-reported data, we adopted a sliding average method to process the number of reported cases per day. The C*_t_* denotes the number of reported cases on that day and the data in the time range (t-n, t+n) were summed and averaged as the number of reported cases on that day, where n denotes the number of days in interval t and 2n+1 denotes the time range. In our study, n was used as 3, i.e., 7 days as the time window for the sliding average. The exact formula is as follows:


$${\text{C}}_{t,\;n}\;=\frac{{\text{C}}_{\text{t}-\text{n}}+{\text{C}}_{\text{t}-\text{n}+1}+{\text{C}}_{\text{t}-\text{n}+2}+\ldots +{\text{C}}_{\text{t}}+\ldots +{\text{C}}_{\text{t}+\text{n}-2}+{\text{C}}_{\text{t}+\text{n}-1}+{\text{C}}_{\text{t}+\text{n}}}{2\text{n}+1}$$

### 2.3 Assessment of transmissibility

#### 2.3.1 Compartment model

**Figure 1:**
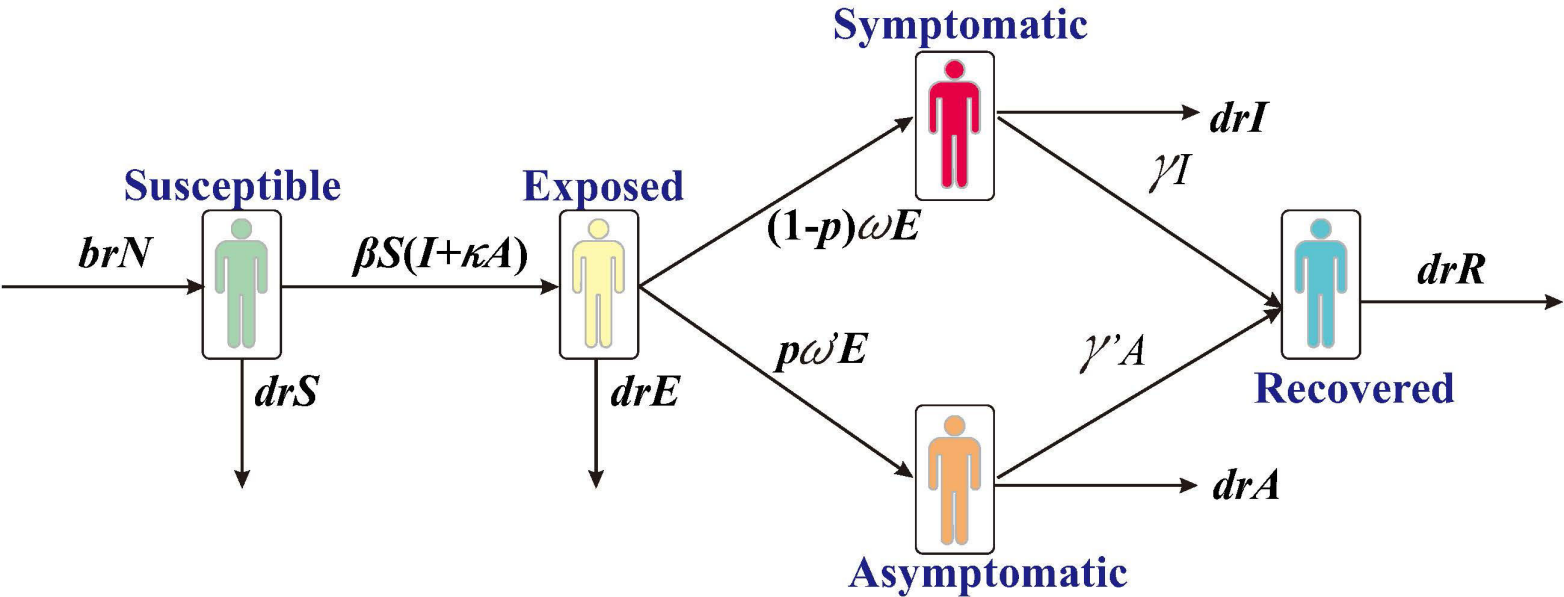
Framework of the SARS transmission dynamics model.

In this study, we have constructed a SEIAR transmission dynamics model according to the natural history of SARS, which divides the individuals into five compartments: S, susceptible; E, exposed; I, infected; A, asymptomatic; R, removed (**Figure 1**).

The model was developed with the following assumptions:

1) Assume that the birth and death rates of the population are *br* and *dr*, respectively. The number of individuals born who are all susceptible is br*N at the time of *t*, where *N* denotes the total population; the number of natural deaths in each compartment are dr*S, dr*E, dr*I, dr*A, and dr*R, respectively.
2) Assume that the coefficient of transmission rate after effective contact between S and I is *β*. Meanwhile, assuming A is infectious and its transmissibility is a *κ* multiple of I (0<*κ*< 1), the number of new infections at moment t is *βS* (*I + κA*).
3) Assume that the proportion of asymptomatic infections is *p* and that the incubation and latency periods are 1/*w* and 1/*w*’, respectively, the number of individuals whose status change from E to A and I at moment t is p*w’*E and (1- p) *w*E, respectively.
4) Assume that the time interval from onset to a first consultation of *I* is 1/*γ*, and the number of people whose status change from I to R at the moment of t is γ*I. Let the mortality rate be *f*, and the number of deaths is f*I at the moment of *t*.

The differential equations for the transmission dynamics model are as follows:


$$\frac{dS}{dt}=brN-\beta S\left(I+kA\right)-drS$$

$$\frac{dE}{dt}=\beta S\left(I+kA\right)-pw^{’}E-\left(1-p\right)wE-drE$$

$$\frac{dI}{dt}=\left(1-p\right)wE-\gamma I-\left(dr+f\right)I$$

$$\frac{dA}{dt}=pw’E-\gamma ’A-drA$$

$$\frac{dR}{dt}=\gamma I+\gamma ’A-drR$$

Parameters were estimated as followings:

The infection rate coefficients (*β*) were obtained by the fitting model. The reported case data were used as the fitted data to fit the model. Based on the prevalence curves of different regions, the prevalence curves were divided into two periods according to the temporal inflection points of the increasing and decreasing parts of the prevalence curves, then they were fitted to the model for assessing the *β* values during the two time periods, *β_1_* and *β_2_*, respectively.

Previous studies reported that the average incubation period of SARS was 6.4 days (Chan-Yeung and Xu, 2003), so *ω=*1/6.4 for all of the regions of our study. However, since the incubation period of SARS in Shanxi Province could not be determined in previous studies, the incubation period in Shanxi Province was assumed to be the same as *ω*’ = 1/6.4. The latency period in Beijing City, Guangdong Province, Hebei Province, Inner Mongolia Autonomous Region, Tianjin City, Hong Kong, and Taiwan Province was 7.6 (Lu et al, 2003), 4.5 (Li LH, 2003), 8 (Zhang Zk and Xu, 2004), 6.67 (Zhang Bin et al, 2008), 4.2 (Li, 2003), 6.37 (Chau and Yip, 2003), and 9 days (Hsueh et al., 2003), respectively, based on existing studies, so *ω*’ of various cities was respectively set to 1/7.6, 1/4.5, 1/8, 1/6.67, 1/4.2, 1/6.37 and 1/9.

Studies have demonstrated that the asymptomatic (*p*) proportion in Tianjin was 18% (Wei et al., 2009), so *p* was set for Tianjin as *p*=0.18. Other studies have reported that the proportion in Hong Kong was 2% (Woo et al., 2004). Therefore, *p* was set as *p*=0.02 for Hong Kong. The value of *p* was set as *p*=0.12 for other regions with an average percentage of asymptomatic SARS of 12% (Wu et al., 2021).

There were no reports showing the exact transmissibility of asymptomatic infections of SARS so that we referenced to influenza and assumed that the transmissibility of asymptomatic individuals was half of that among infected individual (Longini et al., 2005), *κ* = 0.5.

Previous studies have showed that asymptomatic people are infectious during the onset of the disease (Wilder-Smith et al., 2005; Tan, 2006; Tuan et al., 2007), with a duration of approximately 14 days. SARS is infectious at the onset and the duration of the disease in invisibly infected individuals, which is difficult to determine. Therefore, we used the average duration of disease rather than the duration of disease in asymptomatic individuals, *γ* ’ = 1/14 (Lee et al., 2003). According to the literature, the duration of disease is known to be 29 (Wang Yadong et al, 2003), 24.52 (Xia Jielai and Zhang, 2003), 21.52 (Yang Muxiang et al, 2004), 25 (Zhang Bin et al, 2008) and 32.9 (Xu Shi-Xin et al, 2004) in Beijing City, Guangdong, Hebei, Inner Mongolia, and Tianjin City, respectively, while the average duration of disease is 23.7 (Hui and Zumla, 2019). Therefore, for the remaining sites, *γ* values are set to be 1/29, 1/24.52, 1/21.52, 1/25, 1/32.9 and 1/23.7.

Due to the high mortality rate of critically ill SARS patients, and the variation in the mortality rate (*f)* varies from region to region, according to the literature as well as reports, the *f* was 7.66%(Chen Q et al, 2004), 8.00%(Xu Ying, 2004), 6.10%(Liu F et al, 2006), 8.00%(Zhang Bin et al, 2008), 5.35%(Liu L et al, 2005), 3.64%(Peng Gw et al, 2003), 17.00%(Organization, 2015) and 10.70%(Organization, 2015).

Birth rate and death rate of the population were obtained from statistical yearbooks of the eight regions. The annual birth rates of each area were 0.00506, 0.00714, 0.01143, 0.0092, 0.01226, 0.01366, 0.007, and 0.0106. The annual mortality rates were 0.00515, 0.00604, 0.00627, 0.0062, 0.000622, 0.00531, 0.0055, and 0.0058. Since the simulation time in the study is in days, for the parameter *br* and *dr*, the annual birth rate was divided by 365 days. The detailed information about the parameters in the model are given in **Table 1**.

**Table 1:** Cumulative number of SARS cases and duration of the outbreak in different regions.

Parameter	description	unite	regions								source
			Beijing	Tianjin	Hebei	Inner Mongolia	Shanxi	Guangdong	Hongkong	Taiwan	
*β*1	Transmission relative rate of upwards period	day^-1^	–	–	–	–	–	–	–	–	Curve fitting
*β*2	Transmission relative rate of descend period	day^-1^	–	–	–	–	–	–	–	–	Curve fitting
*κ*	Relative transmissibility rate of asymptomatic to symptomatic individuals	1	0.5	0.5	0.5	0.5	0.5	0.5	0.5	0.5	Assume
*p*	Proportion of the asymptomatic	1	12%	18%	12%	12%	12%	12%	2%	12%	References [26-28]
ω	Incubation relative rate	day^-1^	1/6.4	1/6.4	1/6.4	1/6.4	1/6.4	1/6.4	1/6.4	1/6.4	References [18]
*ω’*	Latent relative rate	day^-1^	1/7.6	1/4.2	1/8	1/6.67	1/6.4	1/4.5	1/6.37	1/9	References [18-25]
*γ*	Recovery rate of the infectious	day^-1^	1/29	1/32.9	1/21.52	1/25	1/23.7	1/24.52	1/23.7	1/23.7	References [22, 34-38]
*γ’*	Recovery rate of the asymptomatic	day^-1^	1/14	1/14	1/14	1/14	1/14	1/14	1/14	1/14	References [33]
*f*	Mortality	1	7.66%	8.00%	6.10%	8.00%	5.35%	3.64%	17.00%	10.70%	References [22, 39-44]
*br*	Population birth rate in 2003	1	0.00506	0.00714	0.01143	0.0092	0.01226	0.01366	0.007	0.0106	Statistical Yearbook
*dr*	Population death rate in 2003	1	0.00515	0.00604	0.00627	0.0062	0.000622	0.00531	0.0055	0.0058	Statistical Yearbook
*N*	The population number in 2003	1	14564000	10113000	67520000	23858000	33142900	79542200	6730800	22604000	Statistical Yearbook

#### 2.3.2 Calculation of *R*_0_

The basic reproduction number (*R*_0_) is generally used to evaluate the transmissibility of infectious disease, which was defined as the number of new cases that can be expected to be generated during the infectious period of one case imported into a susceptible population (Chen et al., 2014; Chen et al., 2016). When *R*_0_ > 1, SARS spreads epidemic; when *R_0_* < 1, the epidemic tends to be controlled; when *R_0_ =*1, the epidemic is not expanding and does not end simultaneously. *R*_0_ = 1 is the threshold for the prevalence of infectious diseases in the ideal state. In a situation where the population is not fully susceptible or where interventions are implemented, the transmissibility of an infectious disease should be quantified by effective reproduction number (*R_eff_*). It has been proposed that in an SEIAR model with population births and deaths, the limitation of *R_eff_* must be calculated as follows (Huang et al., 2019; Zhao et al., 2020):


$$\underset{dr\to \infty }{\lim}R_{eff}=\beta S\left(\frac{1-p}{\gamma +f}+\frac{p}{\gamma^{’}}\right)$$

#### 2.3.2 Calculation of *R_t_*

In the real-world, contact rates and transmissibility may vary over time, particularly with the implementation of control measures. therefore, we also used time-varying reproduction number (*R_t_*) which represents the mean number of secondary cases that an infected individual would transmit in the time of t to quantify the transmissibility of SARS (Cori et al., 2013), serial interval (*SI* = 8.4 ± 3.8) for symptom generations in each city(Lipsitch et al.), and calculated *R_t_* values using seven days as a time range. Reference was made to Cori et al. (Cori et al., 2013) using EpiEstim package (version 2.2.4) in R software (version 4.1) to calculate *R_t_*, which is calculated as follows: propagation is assumed to be constant over a time period of [t−τ+1;t] and is measured with $R_{t,\tau }$


$$I_{t}\;\sim \;Possion\left(R_{t}\sum_{s=1}^{t}I_{t-s}\omega_{s}\right)$$

$$P(I_{t}?I_{0},\ldots ,I_{\left(t-t\right)},\omega ,R_{t})=\frac{{\left(R_{t}\Lambda_{t}\right)}^{It}e^{Rt\Lambda t}}{I_{t}!}$$

$${\text{Λ}}_{t}=\sum_{s=1}^{t}I_{t-s}\omega_{s}$$

$$P\left({\text{I}}_{\text{t}-t+1},\ldots ,{\text{I}}_{\text{t}}{\text{|I}}_{0},\ldots ,{\text{I}}_{\text{t}-t},\text{ω },R_{\text{t},t}\;\right)=\;\prod_{s=t-\tau +1}^{t}\frac{{\left(R_{t,\tau \;}{\text{Λ}}_{s}\right)}^{I_{s}}e^{-R_{t,\tau \;}{\text{Λ}}_{s}}}{I_{s}!}$$

$$P\left({\text{I}}_{\text{t}-\text{t}+1},\ldots ,{\text{I}}_{\text{t}},R_{\text{t},\text{t}}|{\text{ I}}_{0},\ldots ,{\text{I}}_{\text{t}-\text{t}},\text{ω }\right)=P\left({\text{I}}_{\text{t}-\text{t}+1},\ldots ,{\text{I}}_{\text{t}},R_{\text{t},\text{t}}{\text{| I}}_{0},\ldots ,{\text{I}}_{\text{t}-\text{t}},\text{ω},{\text{ R}}_{\text{t},\text{t}}\;\right)\;P\left(R_{t,t}\right)$$

#### 2.3.3 Model simulation and statistical methods

The model was simulated with Berkeley Madonna 8.3.18 software, and the differential equations were solved using the fourth order Runge-Kutta method. The output of the model-fitted data was determined based on the least root mean square (LRMS), and the optimal output was correlated by R version 4.2.1 (http://www.sthda.com/english/wiki/correlation-test-between-two-variables-in-r) for correlation analysis The formula is as in (a), and the formula for the t-test statistic with the correlation coefficient r is as in (b), the closer correlation coefficient is to 1, the better the model fit is indicated by *P*< 0.05.


(a) $$r=\frac{\sum^{}\left(x-m_{z}\right)\left(y-m_{y}\right)}{\sqrt{\sum^{}{\left(x-m_{x}\right)}^{2}\sum^{}{\left(y-m_{y}\right)}^{2}}}$$

(b) $$t=\frac{r}{\sqrt{1-r^{2}}}\sqrt{n-2}$$

### 2.4 Statistical analysis

To minimize the interference with noise and data validity of *R_t_* calculation due to centralized reporting and processing of WHO-reported data, we adopted a sliding average method to process the number of reported cases per day. The transmissibility of an infectious disease was quantified by an effective reproduction number (*R_eff_*). It has been proposed that in SEIAR model that includes population births and deaths, limitation of *R_eff_* could be estimated by excel, as in previous studies (Huang et al., 2019; Zhao et al., 2020). We used the time-varying reproduction number (*R_t_*) to quantify the transmissibility of SARS by R version 4.2.1. The model was simulated with Berkeley Madonna version 8.3.1, and the differential equations were solved using the fourth order Runge-Kotta method.

In this study, the statistical analysis in the number of infections between male and female, whether the *R*_0,_ *R_eff_* and *R_t_* were smoothed, comparison of *R_t_* of rising phase with *R*_0_, and comparison of *R_t_* in the decreasing phase with *R_eff_* were analyzed by IBM Statistic SPSS (version 13.0, IBM Corp., Armonk, NY, USA). We took *P*<0.05 to be significant, *P*< 0.001 to be statistically significant.

## 3 Results

### 3.1 Epidemiological characteristics of SARS in China

**Figure 2:**
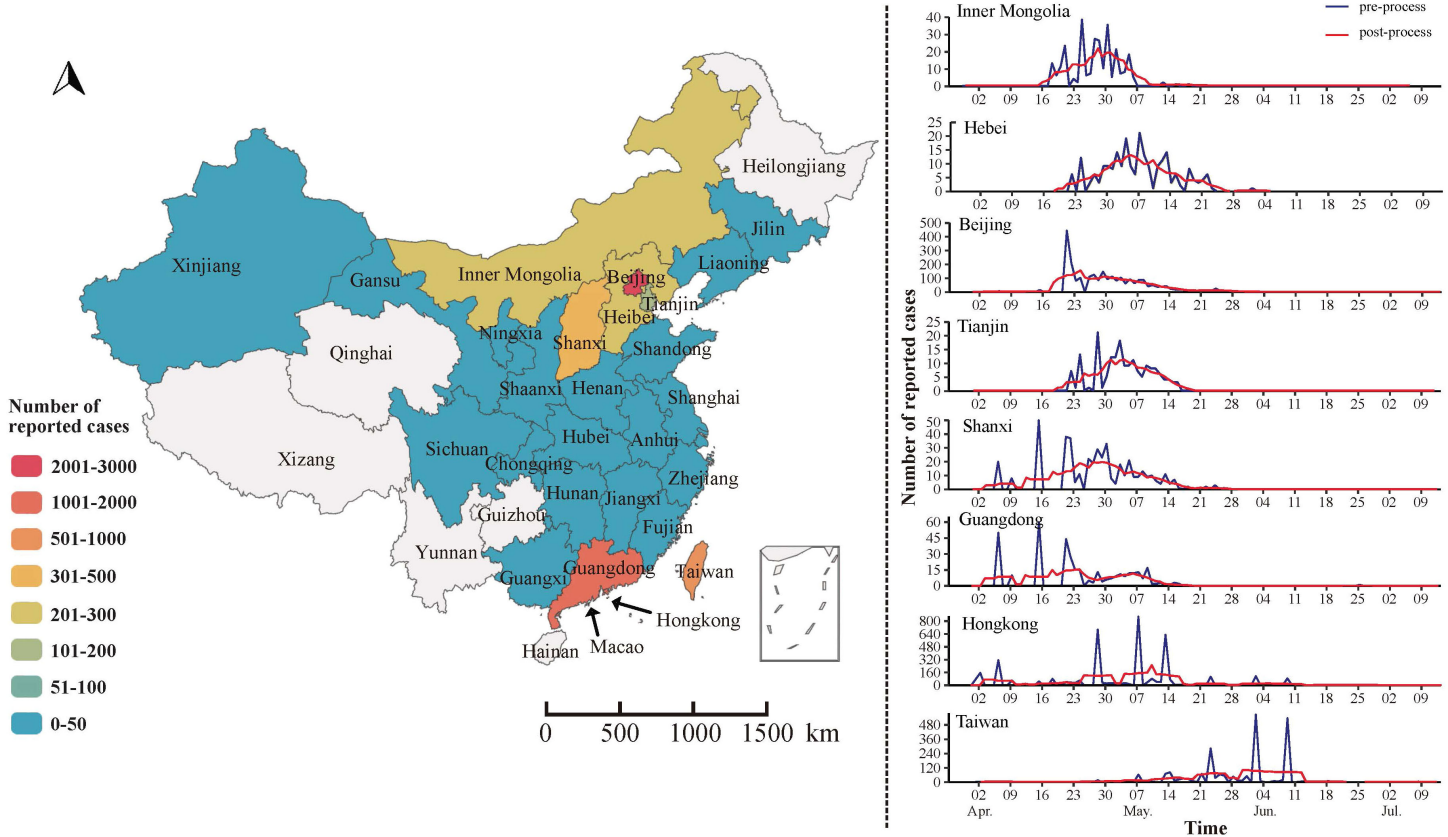
Spatial and temporal distribution of SARS in China.

From April to July, 2003, Beijing, Guangdong, and Hong Kong became the core areas of SARS transmission. The distribution pattern of SARS showed a gradual expansion from these regions to others. Among them, Beijing, Guangdong, Hong Kong, and Taiwan experienced more severe outbreaks, with over 1,000 reported cases each, and Hong Kong and Taiwan experienced SARS epidemics lasting over 90 days, while the remaining 18 provinces reported no more than 50 cases of SARS (**Figure 2**).

**Figure 3:**
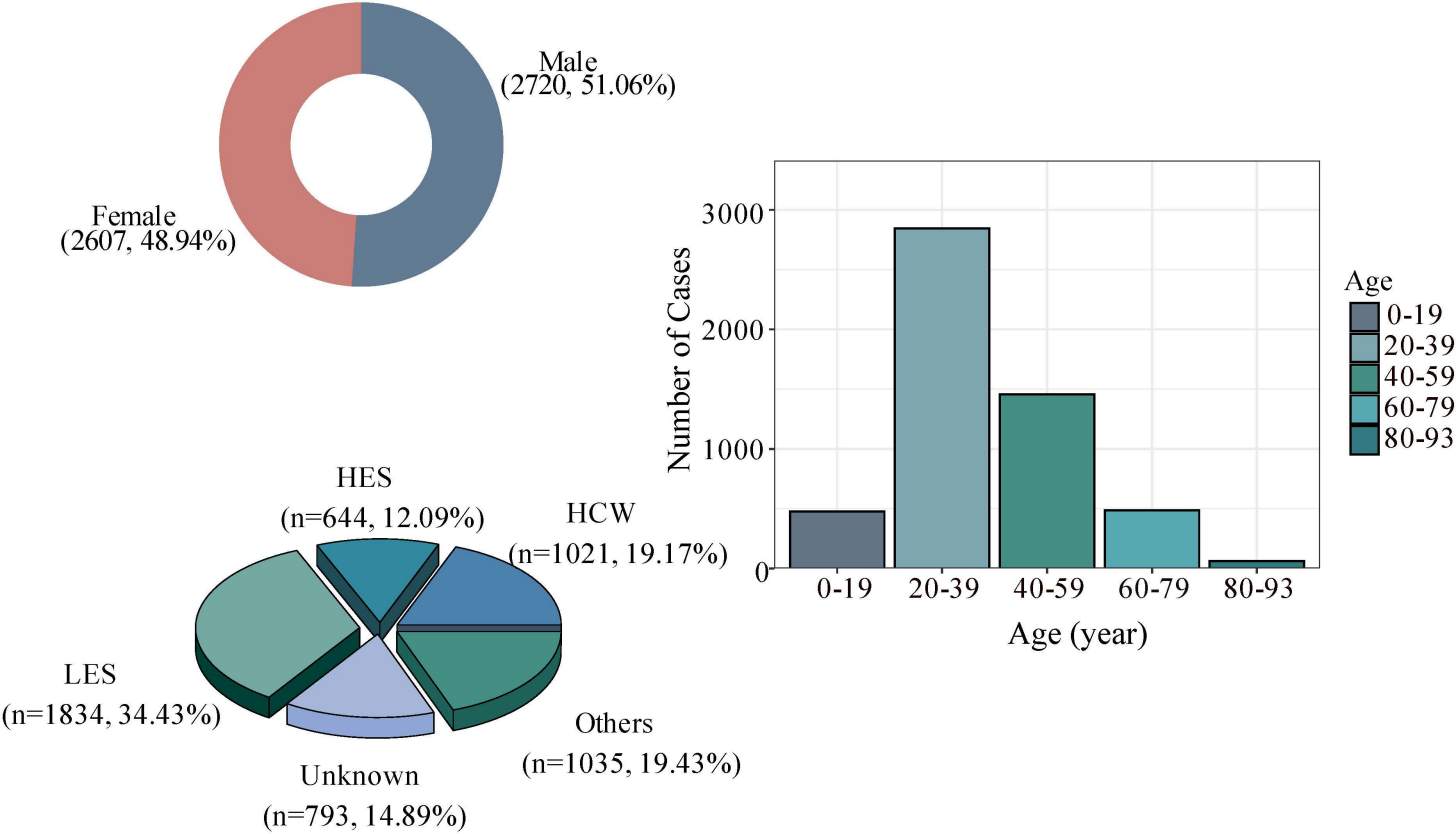
Basic characteristics of SARS cases in China; HCW, Health care worker. HES, High economic status, such as businessmen, civil servants, and teacher. LES, Low economic status such as farmers, day care workers/nannies, waiters/waitresses and workers. Others: three groups closely related to age, namely pensioners, students, and children.

Further analysis of 5327 SARS cases in China found that 343 patients died in the outbreak, with a case fatality rate of 6.4%. The number of reported cases in males and females was 2720 and 2607, respectively, mainly concentrated in the 20-39 age group, followed by the 40-59 age group, which accounted for more than 50%. In addition, the number of reported cases in the low economic status group was 1834. The proportion was 34.43%, followed by those with high economic status (such as businessmen, civil servants, and teachers), the number of reported cases was 1021 (19.17%), and the remaining 644 (12.09%) cases were reported as medical workers (**Figure 3**).

### 3.2 Transmissibility evaluation and analysis

#### 3.2.1 SEIAR dynamics model construction

**Figure 4:**
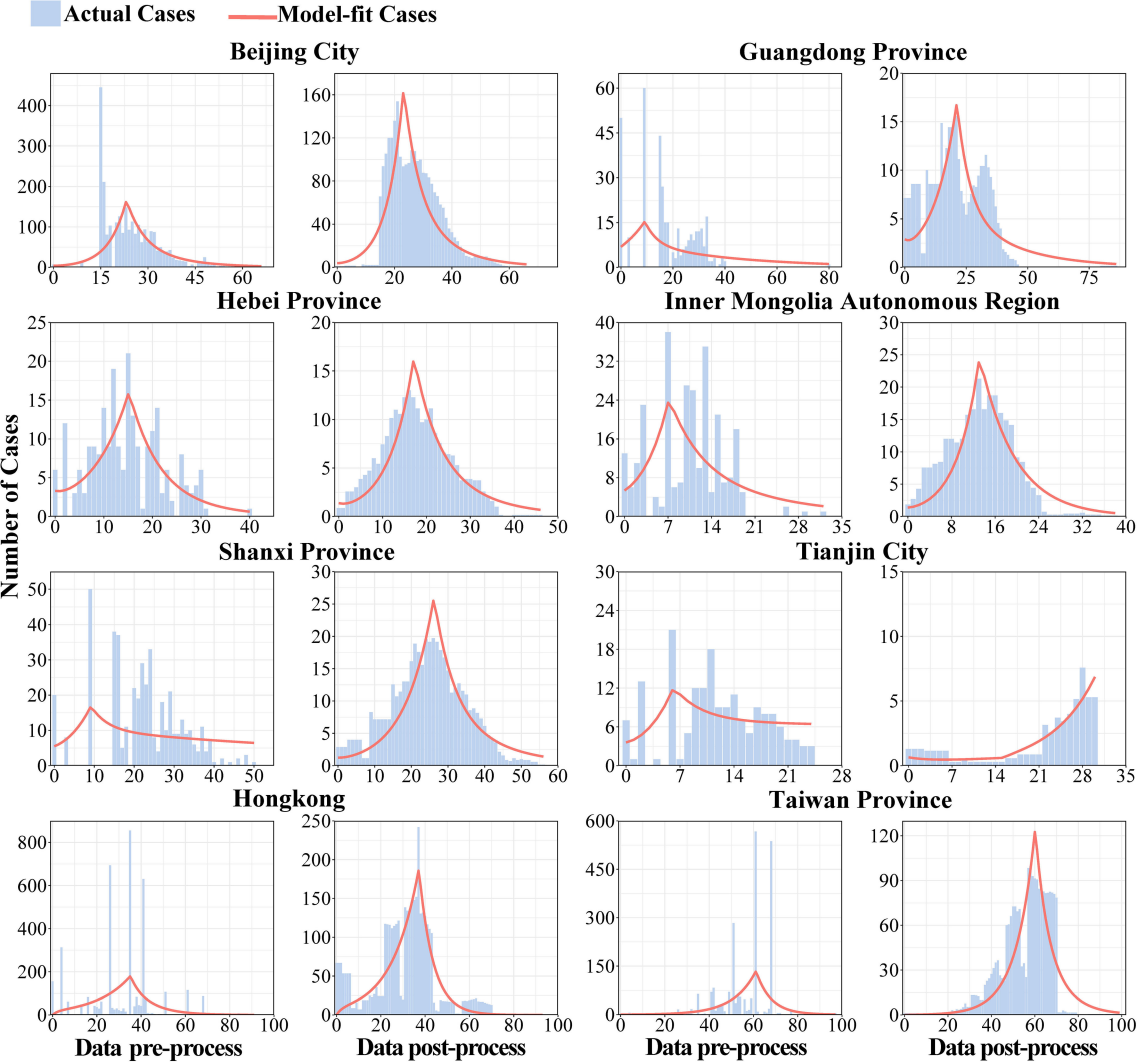
Original-data and smoothed-data of model fitting.

**Figure 5:**
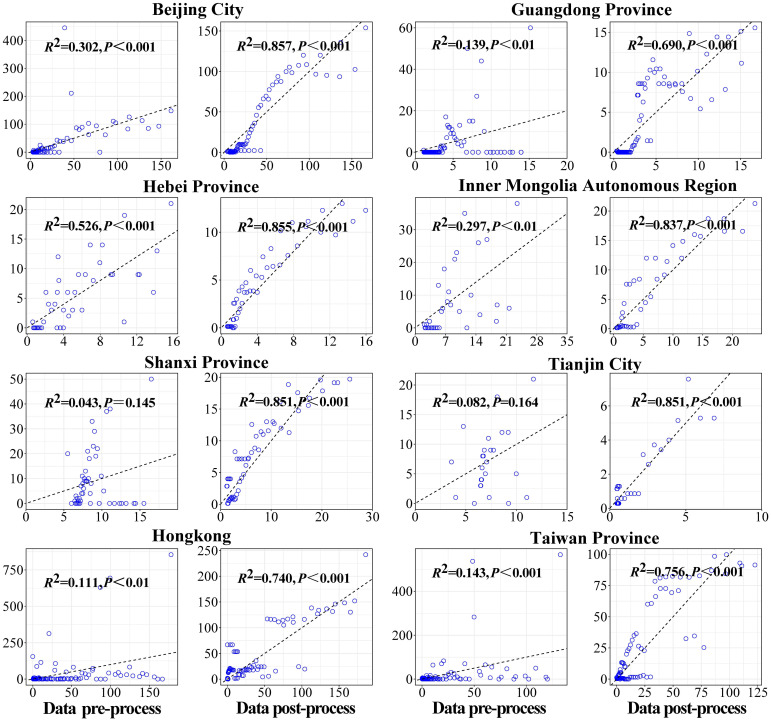
Results of statistic-test of model fitting.

Firstly, the data of key areas were smoothed, and the second and the fourth columns of **Figure 4** showed the results of reported cases in key areas. The number of reported cases of SARS in 8 regions of China in 2003 and the statistical tests for model fitting are shown in **Figure 5**. Correlation analysis of the fitted values with the actual number of reported cases showed that the mean value of *R* ^2^ was 0.205 ± 0.160 when fitted using the original data reported by WHO. Meanwhile, after smoothing the data, the mean value of *R* ^2^ was 0.738 ± 0.082, indicating that the model fitting effectiveness was better after the data smoothing was processed (*P*<0.001) (**Supplementary Table 1**).

#### 3.2.2 Analysis results of SARS transmissibility

According to the calculation results of the transmissibility index based on the original data, the *R*_0_ of SARS in descending order was: Tianjin City (*R*_0_ = 8.249), Inner Mongolia Autonomous Region, Shanxi Province, Hebei Province, Beijing City, Guangdong Province, Taiwan Province, and Hong Kong. The transmissibility of SARS was quantified by *R_eff_* and the values of above cities were all lower than 1, which demonstrated that comprehensive measures taken since the outbreak of SARS were effective and blocked the transmission of SARS virus successfully.

In Guangdong, the time to *R_eff_* <1 was day 7 after the start of the epidemic. In Inner Mongolia, the rising stage of the epidemic was day 13, followed by the alert for the control phase. In Tianjing, the time from the natural transmission phase to the controlled phase of the epidemic was 6 days. The longest response time was in Taiwan (60 days).

After smoothed, the *R*_0_ of Inner Mongolia and Tianjin was the highest. The transmissibility, described via *R*_0_ in descending order were Inner Mongolia, Tianjin, Beijing, Hebei, Shanxi, Guangdong, Taiwan, and Hong Kong. Among those, *R*_0_ of Shanxi, Tianjin was less than the original data, while they were greater compared with that of the remaining cities. *R*_0_ in some cities such as Hebei fluctuated greatly, which was from 4.154 to 5.821. For all 8 cities, they all had *R_eff_* less than 0.5, which was consistent with the pre-processed data output, and the *R_eff_* in Inner Mongolia and Hongkong was less than 0.001.

There was no significant difference in the median of *R_t_*, *R*_0_, *R_eff_*, whether they were smoothed or not (*P*>0.05), indicating that the model was relatively stable. And the smoothing of the data only changed the distribution of SARS cases, not its transmissibility across cities (**Supplementary Tables 2** , **Table 3**).

### 3.3 *R_t_* Analysis

**Figure 6:**
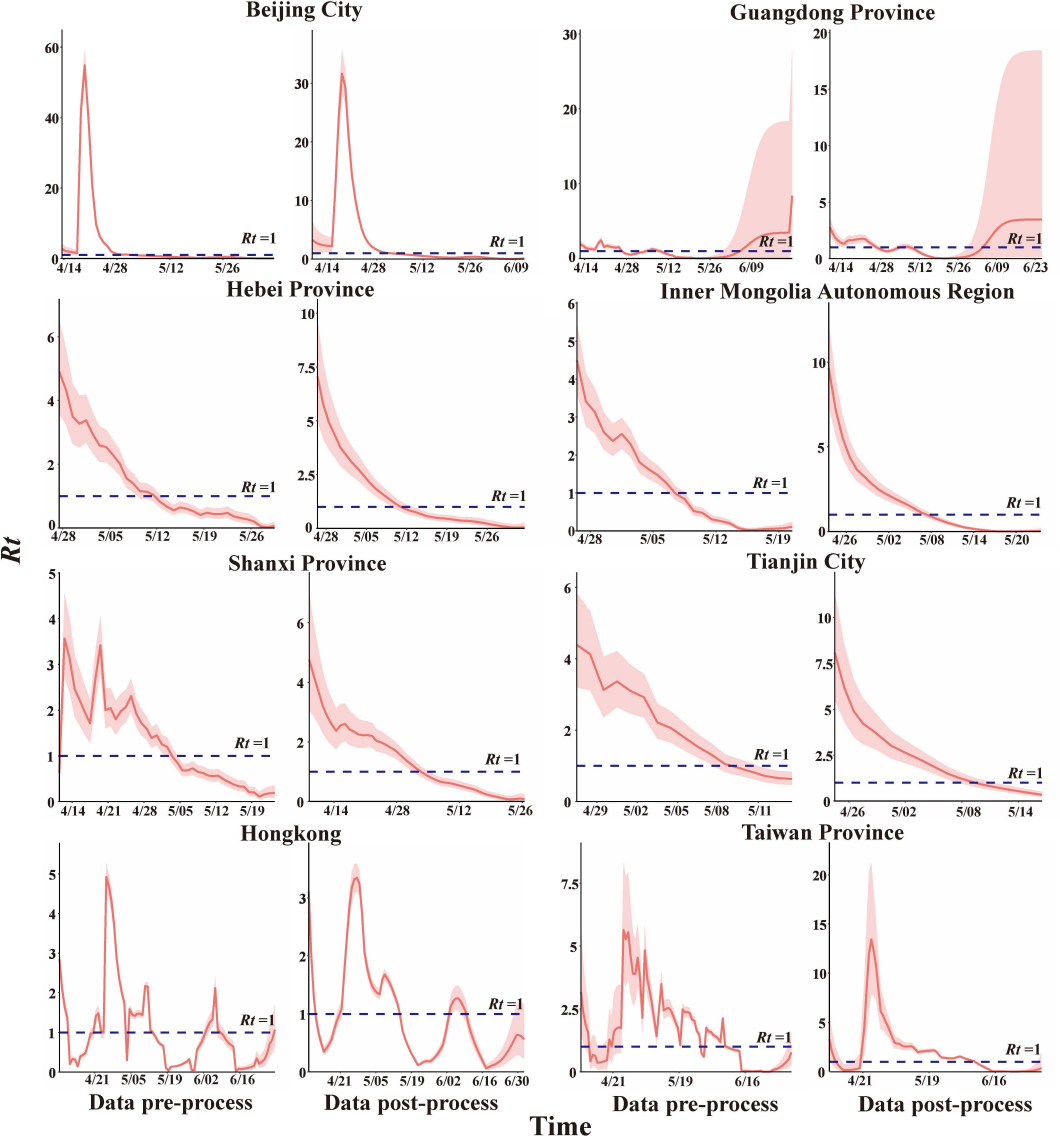
The value of Rt in different regions.

The smoothed data were used to estimate *R_t_* (**Figure 6**). The peak was observed in Beijing, *R_t_* = 29.387, here is lasted 11 more days. However, the outbreak still was controlled within 25 day which is shorter than in the actual situation. *R_t_* in Hebei, Inner Mongolia, Shanxi, and Tianjin showed an overall decreasing trend with peaks of *R_t_* = 7.065, 9.653, 4.760, and 8.085, respectively, and *R_t_* fell below 1 on day 16, 15, 26, and 16 respectively. *R_0_* dropped to *R_eff_* one day earlier in Hebei and Tianjin and two days later in Inner Mongolia.

*R_t_* in Guangdong Province and Hong Kong showed a fluctuating trend. During the epidemic in Guangdong, there were 42 days with an *R_t_* higher than 1. *R_t_* =1.63 (IQR: 1.323-1.744), which was less than *R_0_* (*R_0 _=*3.223), and cumulatively, there were 36 days with an *R_t_* lower than 1, the average *R_t_* = 0.630 ± 0.068, which was consistent with the results of *R_eff_*. The Hong Kong epidemic had its *R_t_* values higher than 1 for a total of about 35 days, *R_t_* =1.996 (IQR: 1.284-3.108), lower than *R_0_* (*R_0_=*2.159), when *R_t_* <1, the average *R_t_* =0.539 ± 0.062 in descend stage, consistent with *R_eff_*. *R_t_* in Beijing and Taiwan showed single-peak fluctuations, peaking at 29.387 and 13.417 at day 11 and 18 respectively after the outbreak. More importantly, it was a total of 40 days had *R_t_* < 1 during the epidemic in Taiwan (**Supplementary Table 4**).

*R*_0_ was generally higher than *R_t_* (*P*<0.05) during the epidemic escalation phase, and *R_eff_* was higher than *R_t_* (*P*<0.05) after the epidemic was controlled (**Tables 2**, **3**).

**Table 2:** Comparison of the transmissibility calculated in original data.

Region	original data							
	*R*_0_	*R_t_* (ascending stage)	*R*_0_ - *R_t_*	*P*	*R_eff_*	*R_t_* (descent stage)	*R_eff_* - *R_t_*	*P*
Beijing City	5.667	2.753	2.914	0.003	0.164	0.347	-0.184	0.003
Guangdong Province	3.232	1.525	1.707		0.552	0.960	-0.408	
Hebei Province	4.154	2.939	1.215		0.060	0.475	-0.415	
Inner Mongolia	7.396	3.415	3.981		0.238	0.501	-0.263	
Shanxi Province	4.612	2.458	2.154		0.827	0.79	0.037	
Tianjin City	8.249	4.257	3.992		0.986	1.534	-0.584	
Hongkong	1.863	1.418	0.445		0.180	0.645	-0.465	
Taiwan Province	2.808	2.037	0.771		0.009	0.170	-0.161	

**Table 3:** Comparison of the transmissibility calculated in smoothing data.

Region	Smoothing Data							
	*R*_0_	*R_t_* (ascending stage)	*R_0_* - *R_t_*	*P*	*R_eff_*	*R_t_* (descent stage)	*R_eff_* - *R_t_*	*P*
Beijing City	6.319	5.064	1.255	0.002	0.359	0.358	0.001	0.013
Guangdong Province	3.974	1.623	2.531		0.320	0.865	-0.545	
Hebei Province	5.821	3.401	2.420		0.134	0.4127	-0.279	
Inner Mongolia	8.018	3.648	4.370		0.000	0.227	-0.227	
Shanxi Province	4.349	2.294	2.055		0.189	0.468	-0.279	
Tianjin City	7.868	4.218	3.650		0.168	1.056	-0.888	
Hongkong	2.159	1.481	0.700		0.000	0.460	-0.460	
Taiwan Province	3.223	2.127	1.096		0.102	0.136	0.340	

### 3.4 Sensitivity analysis of key parameters

**Figure 7:**
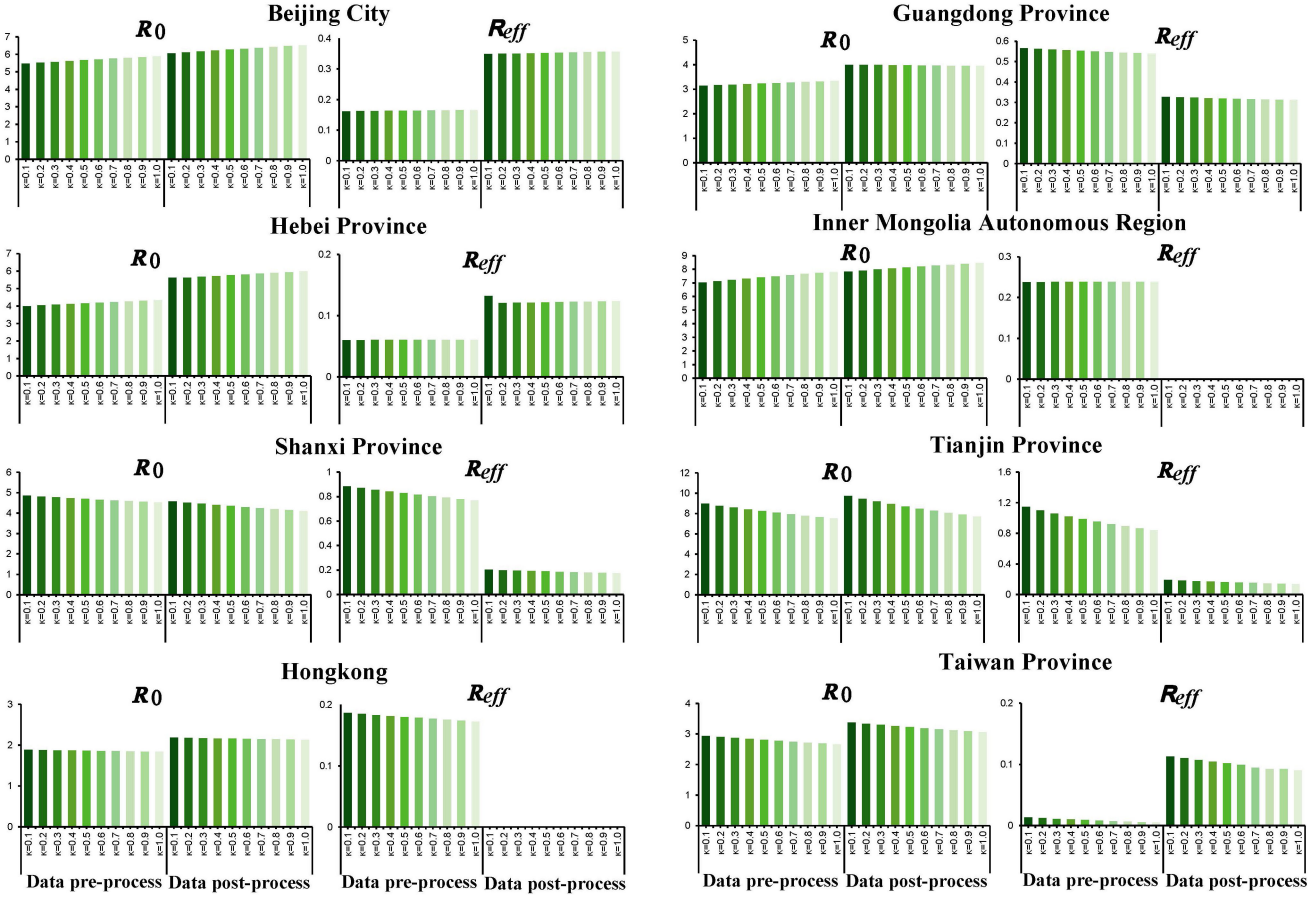
Sensitivity analysis of the key parameter κ for model.

The research results (**Figure 7**) indicated that the *R*_0_ of SARS outbreak in Beijing City, Hebei, and Inner Mongolia increases as the infectivity of asymptomatic infections strengthened, while that in Shanxi, Tianjin City, Hong Kong, and Taiwan, it decreases. The *R*_0_ of Guangdong, showed inconsistency before and after smoothing, suggesting that the transmission of asymptomatic infections could influence the magnitude of SARS spread. The impact of the infectivity of asymptomatic infections on Tianjin City is found to be substantial, with the range of *R*_0_ varying from 7.703 to 9.723. During the epidemic control phase, the transmission of asymptomatic infections has a relatively minor effect on the spread of SARS (**Figure 7**). Therefore, incorporating the transmission of asymptomatic infections could influence the transmissibility of SARS-CoV, and further efforts are needed to obtain more accurate *κ* values to represent the infectivity of asymptomatic infections and restore the true transmissibility of the virus.

## 4 Discussion

In the absence of a comprehensive comparative analysis of the transmissibility of the 2003 SARS epidemic in mainland China, we collected public and publicly available information from several Chinese provinces and regions, including Hong Kong and Taiwan, where the outbreak was particularly severe. We employed data smoothing techniques to restore the actual epidemic curves as accurately as possible, and applied both epidemiological models and the *R_t_* to assess the transmissibility of these regions. We compared and analyzed the differences in results obtained from different methods, as well as the disparities in transmissibility among different regions.

The SARS epidemic initially emerged in Guangdong and subsequently spread outward from the core areas, including Guangdong, Hong Kong, and Beijing. In the early stages, aggregated infections due to crowding, confinement, hospital visits, family gatherings, and inadequate precautions in public places were associated with the high incidence in Guangdong (Li Lh et al, 2003). In addition, the proliferation of SARS was further exacerbated by the concomitant occurrence of low levels of solar irradiance and elevated atmospheric moisture during the period spanning January through April (Zhang Ruoling and Yang, 2006). The SARS outbreak in Hong Kong was due to the unique subterranean drainage system, architectural layout, and inherent deficiencies within the healthcare infrastructure of Hong Kong explains more satisfactorily the epidemic propagation of SARS in the region (Lee, 2003). With the arrival of the Spring Festival travel peak, SARS further disseminated nationwide. In Beijing, where the transportation infrastructure is well developed. the distribution of SARS cases in the suburbs which were mostly concentrated in county towns surrounding provincial highways and interprovincial expressways. In the urban areas, a clustered pattern of cases was observed, with the highest concentration occurring at the boundary zone between the northern Third Ring Road and the Chaoyang and Tongzhou districts. Notably, a higher number of cases were imported to Hebei and Shanxi provinces, while Inner Mongolia and Tianjin also experienced secondary transmission (Fang et al., 2009; Cao et al., 2011; Xu et al., 2014). These findings indicate that population movement played a critical role in the rapid transmission of SARS in China.

The true prevalence curves of quantities may differ significantly from the cases and onset dates reported in the study, hence the smoothing method used in this study to minimize the bias caused by centralized reporting. The differences in *R*_0_ and *R_eff_* across different locations mainly stem from regional heterogeneity and varying interventions implemented in each area.

The *R*_0_ of the SARS epidemic was generally higher in Mainland China compared to Hong Kong and Taiwan. *R*_0 Mainland China_=6.058 ± 1.703, *R*_0 Hongkong_ = 2.159, *R*_0 Taiwan_ = 3.223. The *R*_0 Mainland China_ was higher than previous studies on account of a lack of studies on SARS in Mainland China and lacked accurate conclusions. (**Supplementary Table 5**) *R*_0_ in Hongkong and Taiwan was consistent with previous studies, which ranged from 1 to 5 (Hethcote, 2000a; Lloyd-Smith et al., 2003). *R*_0_ in Hong Kong was lower than that of the infection network established by the stochastic simulation model, estimated at *R*_0 _= 3.6(IQR: 3.1-4.2) in Hong Kong (Wallinga and Teunis, 2004). However, the model cannot simulate the natural history of SARS transmission. Furthermore, an SI model estimated an *R*_0_ of 1.7 (0.44-2.29) for Hong Kong (Chowell et al., 2004), the differences may be because of the use of SI model that considers only basic interpersonal transmission characteristics.

All regions have adopted similar preventive and control measures, including but not limited to travel restrictions, quarantine, contact tracing, etc. therefore, succeeded in epidemic control (*R_eff_* <1). However, there were significant differences in the timing of epidemic control, different levels of epidemic prevention led to different SARS transmissibility in each city (Chan-Yeung and Xu, 2003). *R*_0_ in Inner Mongolia and Tianjin fell below 1 in a relatively short period of time and the outbreak was relatively mild. They have taken effective measures at the beginning of the outbreak and no infection was reported in local hospitals. Furthermore, patients seeking medical treatment in Beijing during the SARS epidemic helped to alleviate the local healthcare pressure, indirectly benefiting the affected regions (Sciences C.A.O, 2003).

Overall, *R_t_* in all regions showed a downward trend, but there were significant fluctuations in Guangdong, Hong Kong and Taiwan compared to other areas. Guangdong responded quickly and was able to contain the outbreak in a short period of time. However, China did not take continuous control measures for a period of time, which facilitated domestic and international transmission and the rebound and fluctuation of the outbreak in Guangdong (Ahmad et al., 2009). In Hong Kong, which displays both a high population density and a high mobility, the economy centers are close to the Pearl River Delta, making it the main area where SARS continues to appear in South China. In addition, Hong Kong adopted a different healthcare system than Mainland China, with many private services and inadequate ventilation system facilities, which led to the second outbreak (Naylor et al., 2004). *R_t_* in Hong Kong showed a fluctuating trend, with nosocomial infection in March and its spread affecting many medical staff and students. The second outbreak began with hospital-to-community transmission on April 15, 2003. The third phase corresponded to new cases which continued to be reported but showed a decreasing trend from early May to mid-June 2003 (Law et al., 2020). After the first case was confirmed in Taiwan on March 14, the government implemented quarantine, and effectively controlled the epidemic (*R_t_* <1). However, from April 23 to May 8, the increase in new cases made the hospital system unable to completely treat all patients in the negative pressure ward (Chen et al., 2005). So, the epidemic rebounded, and *R_t_* showed a volatility trend. The *R_t_* value of Beijing was high and abnormal compared to other cities. The possible reason was that Beijing has a large population baseline and has two clustered spaces in urban and suburban areas. At the same time, the government did not take relevant measures in the early outbreak of SARS, resulting in the *R_t _* of March and April being abnormally higher than that of other cities. But epidemic was controlled after implementing the relevant strategy in mid-April (Shen et al., 2004; Cao et al., 2010). Wallinga et al. assessed the mean value of *R_t _* of Hong Kong, Vietnam, Singapore, and Canada as about 3 using the individual random model, consistent with our study (*R_t_* =2.982) (Wallinga and Teunis, 2004). However, the mean value of Hong Kong was 3.6, which was higher than 1.481 in this article (Wallinga and Teunis, 2004). The reason was supposed that the method was different as the former used the model while our *R_t_* is calculated by mathematical formula.

Several regions, including Guangzhou City, Hong Kong, and Singapore, reported cases of asymptomatic infection during the SARS epidemic, which indicates that the risk of SARS-CoV infection for asymptomatic or mildly symptomatic individuals cannot be ignored. Asymptomatic individuals can transmit the virus and represent a significant yet difficult to detect source of infection. In 2003, the lack of a strict comprehensive nucleic acid testing strategy for all individuals allowed unnoticed asymptomatic infected individuals to spread the disease. Our compartmental model incorporated asymptomatic individuals, and the results showed that the contagious of asymptomatic individuals had an impact on the transmissibility of SARS. The overall *R*_0_ was larger than previous studies, but the contribution of SARS transmission varied in different regions, which may be due to variations in the proportion of asymptomatic and symptomatic individuals in each region as well as societal differences. The current COVID-19 and Middle East Respiratory Syndrome (MERS) epidemics have a higher proportion of asymptomatic individuals than SARS (Al-Tawfiq, 2020; Gao et al., 2021), suggesting that the contribution of asymptomatic individuals to the spread of the epidemic is significant. Evaluating the transmissibility of asymptomatic individuals during the initial stages of the epidemic is meaningful, and implementing monitoring and control measures can reduce the risk of widespread transmission.

Due to the lack of detailed pathogen data, there was no literature analyzing the regional etiological differences of SARS. However, some studies observed the epidemiological parameters, such as symptoms and average incubation period of SARS cases in Beijing, Taiwan, and Hong Kong, differed. So far, no solid studies indicated the significant genomic difference of SARS isolated in different regions in the outbreak in 2003. Therefore, it cannot be ruled out that differences between *R*_0_ and *R_eff_* were caused by the differences in viruses in different regions. The occurrence of SARS cases is highly seasonal, so differences in transmissibility due to climate in different regions cannot be excluded (Lau et al., 2010), among them, temperature and humidity were negatively correlated with SARS (Yuan et al., 2006). With the growing trend of globalization and the increasing frequency of human interactions across international borders, respiratory infectious diseases have emerged as significant challenges to global public health. To date, humans have experienced three episodes of respiratory coronaviruses, SARS-CoV, MERS-CoV, and SARS-CoV-2. These viruses have a common ancestry and exhibit analogous transmission properties. Due to the continuous evolution of the virus, exploring the transmission characteristics and evaluating the prevention and control effect of SARS can provide an essential basis for the understanding as well as prevention and control of the novel respiratory coronavirus in the future. Meanwhile, the regional heterogeneity of transmission characteristics can give a direction for further exploration of the environmental influencing factors of the coronavirus.

## 5 Limitations

The reported cases were collected from WHO over a long period of time, so the raw data were more concentrated, resulting in some errors in the onset times of the cases. In order to fit the model closer to the onset time of the actual cases, we averaged and smoothed the data, and the integral distribution of the processed data was more realistic and less noisy than the original data. According to the sensitivity analysis, this parameter *κ* shows to affect the model, so the coefficient *κ* values of asymptomatic individuals in the real world need to be obtained.

## 6 Conclusion

We adopted sliding average method to process the number of reported cases per day and Used transmission dynamics model SEIAR which considering asymptomatic to evaluate the transmissibility of SARS in China of 8 areas. The SARS epidemic in China showed a trend of spreading from the south to the north, with Guangdong and Beijing City being the central regions, respectively to the periphery. This study provides a supplementary analysis of the transmissibility of asymptomatic carriers of SARS. Considering the transmission of asymptomatic patients, there is a significant difference in the transmissibility between different regions, *R*_0 north_ >*R*_0 south_. In contrast, the SARS epidemic in the central region did not stir a large-scale transmission. There were also significant differences in transmissibility among eight cities, with *R*_0_ significantly higher in the northern region than in the southern region. Although different regions were able to control the outbreak successfully, there were differences in the time taken *R*_0_ to fell below than 1 in different regions, with Tianjin City taking the shortest time and Taiwan taking the longest in there. In the later stages of the epidemic, the duration varied among different regions, with the end of the epidemic being shorter in Tianjin and lasting 59 days in Guangdong. The transmissibility was evaluated using the compartment model, which was relatively more compared to that calculated by the *R_t_*, and the *R_t_* results showed a decreasing trend for all regions overall, but Hong Kong and Taiwan differed from other regions by a significant fluctuation in the middle of the epidemic. The *R_t_* of Beijing and Guangdong also demonstrated anomalous values due to the central reporting of data.

## Data availability statement

The original contributions presented in the study are included in the article/**Supplementary Material**. Further inquiries can be directed to the corresponding authors.

## Author contributions

JR: Conceptualization, data analyze, visualization, writing—original draft; HQ: methodology, data curation, data analyze, visualization, writing—original draft; SZ: data analyze, writing—review and editing; HL: data analyze, writing—review and editing; HW: methodology, data analyze; BA: writing—review and editing; KL: visualization; YZ: data curation; QL: data analyze; KF: visualization; LG: conceptualization, supervision; RF: project administration, supervision; TC: conceptualization, project administration, supervision. All authors contributed to the article and approved the submitted version
