# Supplementary material for: Assessment of transmissibility and measures effectiveness of SARS in 8 regions, China, 2002-2003
Source: Front Cell Infect Microbiol. 2023 Aug 10;13:1212473. doi: 10.3389/fcimb.2023.1212473 (PMC10449464; doi:10.3389/fcimb.2023.1212473)
Supplement: Supplementary file 1 [file Table_1.docx]

Supplementary

| Table1Results of statistic-test of model fit | | | | | | | | | | | | | | | |  | | |  |  |
| --- | --- | --- | --- | --- | --- | --- | --- | --- | --- | --- | --- | --- | --- | --- | --- | --- | --- | --- | --- | --- |
| City | Original Data | | | |  |  | | Smoothed Data | | | | | | | | | *P* | |  |  |
|  | R | | R^2^ | *P* | R^2^ (Mean±S) | |  | | R | | R^2^ | | *P* | | R^2^ (Mean±S) | | |  | | |
| Beijing City | 0.549 | 0.302 | | 0.000 | 0.205  ±  0.160 |  | | 0.926 | | 0.857 | | 0.000 | | 0.738  ±  0.082 | | ＜0.001 | | |  |  |
| Guangdong Province | 0.373 | 0.139 | | 0.001 |  |  | | 0.831 | | 0.690 | | 0.000 | |  |  |  |  |  |  |  |
| Hebei Province | 0.725 | 0.526 | | 0.000 |  |  | | 0.925 | | 0.855 | | 0.000 | |  |  |  |  |  |  |  |
| Inner Mongolia Autonomous Region | 0.545 | 0.297 | | 0.001 |  |  | | 0.915 | | 0.837 | | 0.000 | |  |  |  |  |  |  |  |
| Shanxi Province | 0.207 | 0.043 | | 0.145 |  |  | | 0.922 | | 0.851 | | 0.000 | |  |  |  |  |  |  |  |
| Tianjin City | 0.287 | 0.082 | | 0.164 |  |  | | 0.923 | | 0.851 | | 0.000 | |  |  |  |  |  |  |  |
| Hongkong | 0.334 | 0.111 | | 0.001 |  |  | | 0.860 | | 0.740 | | 0.000 | |  |  |  |  |  |  |  |
| Taiwan Province | 0.378 | 0.143 | | 0.000 |  |  | | 0.867 | | 0.752 | | 0.000 | |  |  |  |  |  |  |  |

Table 2 Result of *R_0_* and *R_eff_* in each 8 cities, China

| City | *R_0_* | | *P* | *R_eff_* | | *P* |
| --- | --- | --- | --- | --- | --- | --- |
|  | Original Data | Smoothed data |  | Original data | Smoothed data |  |
| Beijing City | 5.667 | 6.319 | 0.967 | 0.164 | 0.359 | 0.279 |
| Guangdong Province | 3.232 | 3.974 |  | 0.552 | 0.320 |  |
| Hebei Province | 4.154 | 5.821 |  | 0.060 | 0.134 |  |
| Inner Mongolia  Autonomous Region | 7.396 | 8.018 |  | 0.238 | 0.000 |  |
| Shanxi Province | 4.612 | 4.349 |  | 0.827 | 0.189 |  |
| Tianjin City | 8.249 | 7.868 |  | 0.986 | 0.168 |  |
| Hongkong | 1.863 | 2.159 |  | 0.180 | 0.000 |  |
| Taiwan Province | 2.808 | 3.223 |  | 0.009 | 0.102 |  |

Table 3 *R_t_* statistical-test of pre-process and post-process

| City | *P*  normality test | *P*  non-parameter test |  |
| --- | --- | --- | --- |
|  |  |  |  |
| Beijing City | 0.000 | 0.884 |  |
| Guangdong Province | 0.000 | 0.41 |  |
| Hebei Province | 0.000 | 0.711 |  |
| Inner Mongolia Autonomous Region | 0.000 | 0.803 |  |
| Shanxi Province | 0.000 | 0.876 |  |
| Tianjin City | 0.000 | 0.817 |  |
| Hongkong | 0.000 | 0.659 |  |
| Taiwan Province | 0.000 | 0.924 |  |
|  |  |  |  |

| Table 4 fluctuates widely of *R_t_* in four cities | | | | |  |
| --- | --- | --- | --- | --- | --- |
| City | *R_t_*＞1 | *R_t_*＜1 | *R_t_*＞1 | *R_t_* ＜1 |  |
|  | Normality test (*P*) | | IQR | IQR or Means±S |  |
| Beijing City | 0 | 0.208 | 2.109(1.421,9.797) | 0.547±0.057 |  |
| Guangdong Province | 0.037 | 0.073 | 1.613(1.323,1.744) | 0.630±0.068 |  |
| Hongkong | 0.035 | 0.516 | 1.996 (1.284, 3.108) | 0.539±0.062 |  |
| Taiwan Province | 0.03 | 0.031 | 4.638(2.928,8.471) | 0.280(0.153,0.595） |  |

| Table 5 *R*_0_ values of mainland，Hongkong and Taiwan province | | | |  |
| --- | --- | --- | --- | --- |
| Areas | normality test (*P)* | *R*_0_ |  |  |
| Mainland China | 0.432 | （Means±S）6.058±1.703 |  |  |
| Hongkong | —— | 2.159 |  |  |
| Taiwan Province | —— | 3.223 |  |  |
